# Blinatumomab in Relapsed/Refractory Burkitt Lymphoma

**DOI:** 10.3390/cancers15010044

**Published:** 2022-12-21

**Authors:** Jeanne Bohler, Ulrike Bacher, Yara Banz, Raphael Stadelmann, Michael Medinger, Thilo Zander, Thomas Pabst

**Affiliations:** 1 Department of Medical Oncology, University Hospital Inselspital and University of Bern, 3010 Bern, Switzerland; 2 Department of Hematology and Central Hematology Laboratory, University Hospital Inselspital and University of Bern, 3010 Bern, Switzerland; 3 Institute of Pathology, University of Bern, 3008 Bern, Switzerland; 4 Division of Hematology, Department of Oncology, Lausanne University Hospital and University of Lausanne, 1011 Lausanne, Switzerland; 5 Department of Hematology, University Hospital, 4031 Basel, Switzerland; 6 Department of Oncology, Kantonsspital, 6000 Lucerne, Switzerland

**Keywords:** Burkitt lymphoma (BL), relapsed/refractory (r/r), blinatumomab, safety, efficacy, adverse effects (AEs), infections, cytokine release syndrome (CRS), neurotoxicity

## Abstract

**Simple Summary:**

20–40% of patients with Burkitt lymphoma (BL) have relapsing or refractory (r/r) disease, and standard treatment for such patients is poorly established. An unexplored treatment option is the bispecific T-cell engager blinatumomab, as used for the treatment of r/r and minimal residual disease (MRD) positive B-cell precursor acute lymphoblastic leukemia (BCP-ALL). So far, data on the use of blinatumomab in r/r BL are limited. In this retrospective multi-center case series, we investigated blinatumomab treatment in nine patients with r/r BL after 1–3 previous therapy lines. Data on safety and efficacy were collected. No high-grade (≥grade 3) adverse effects (AEs) occurred, and use of blinatumomab was found to be safe. The best response to blinatumomab and survival data varied considerably among patients, but with five from nine patients responding, blinatumomab seems to have activity in patients with r/r BL. Our data suggest that blinatumomab could be further explored in r/r BL.

**Abstract:**

In patients with relapsed/refractory Burkitt lymphoma (r/r BL), overall survival (OS) is poor, and effective therapies and evidence for the best therapy are lacking. The monoclonal antibody blinatumomab may represent a novel option. However, only limited data on the use of blinatumomab in r/r BL are so far available. This multi-center, retrospective case series investigated nine patients with r/r BL treated with blinatumomab. The safety of blinatumomab was assessed with respect to frequency and severity of adverse effects (AEs) infections, cytokine release syndrome (CRS) and neurotoxicity. Progression-free survival (PFS), OS and overall response rate (ORR) were analyzed to assess efficacy. No AEs > grade 2 occurred, and AEs were generally treatable and fully reversible. The best response to blinatumomab was complete remission in 3/9 patients and partial remission in 2/9, whilst 4/9 presented with progressive disease. Median PFS and OS were 2 and 6 months, respectively, ranging from 5 days to 32 months and 11 days to 32 months, respectively. Blinatumomab treatment was a successful bridging treatment to stem cell transplantation in 3/9 patients. The response to blinatumomab varied widely, and only one patient survived longer term, but activity in patients with r/r BL was evident in some patients, with its use being safe, warranting its prospective investigation.

## 1. Introduction

Due to its high proliferation rate, Burkitt lymphoma (BL) is a very chemosensitive disease and the majority of patients can be cured with an intensive chemoimmunotherapy regimen, with 2-year progression free survival (PFS) and overall survival (OS) rates of approximately 60–80% [1,2,3,4,5]. In contrast, patients with relapsed or refractory (r/r) BL, occurring in 20–40% of the patients, have a poor prognosis [6,7,8]. Short et al. analyzed the outcome of adults with r/r Burkitt and high-grade B-cell leukemia/lymphoma (HGBCL). The 1-year OS for the entire cohort was only 11%, with a median OS of 3.7 months (m) in patients with r/r BL [7]. A retrospective review by the European Society for Blood and Marrow Transplantation reported the outcome of patients with relapsed BL who had undergone autologous HSCT for relapsing disease. These results included a 3-year OS of 37% for patients with chemosensitive relapse and only 7% for patients with refractory disease [8].

Due to the poor outcome of r/r BL patients, improved treatment strategies are an unmet need. However, only few studies have evaluated treatment strategies in r/r BL, and evidence for the selection of an optimum therapy in this setting is lacking [6,7]. Currently, treatment for r/r BL is usually salvage chemotherapy together with CD20 antibody treatment. Others report experimental treatment strategies with PI3K, CDK6 and MYC inhibitors, chimeric antigen receptor T-cells (CAR T-cells) or monoclonal antibodies, followed by autologous or allogeneic HSCT for responding patients [7,9,10]. The bispecific T-cell engager blinatumomab (Blincyto^®^, Amgen, Thousand Oaks, CA, USA) might be another novel treatment option. It combines dual binding specificity for CD19, expressed on B-cells, and the CD3 subunit of the T-cell receptor. Thus, it engages and activates cytotoxic T-cells for the redirected lysis of B-cells and can, therefore, be used for treating a variety of aggressive B-cell malignancies [11,12]. To date, blinatumomab is approved for the treatment of r/r B-cell precursor acute lymphoblastic leukemia (BCP-ALL), and for minimal residual disease (MRD) positive BCP-ALL [12]. Common adverse effects (AEs) of blinatumomab are infections, pyrexia, and neutropenia, and also, notably, cytokine release syndrome (CRS) and neurotoxicity [13,14].

Given the strong CD19 positivity of BL cells, blinatumomab should also be effective against BL cells [11,15]. So far, two small studies have reported experiences with blinatumomab in BL cells. The in vitro activity of blinatumomab against rituximab-sensitive and resistant BL and primary mediastinal B-cell lymphoma cell lines was demonstrated, when Burkitt cells were incubated with blinatumomab and T-cells for four hours, and cytotoxicity and cytokine secretion were measured [16]. The authors concluded that blinatumomab significantly enhances T-mediated in vitro cytotoxicity and cytokine secretion against Burkitt cells and that it should, therefore, be investigated as immunotherapy in patients with r/r Burkitt lymphoma [16]. Duell et al. reported three patients with r/r BL, refractory to first salvage chemotherapy and treated with blinatumomab [17]. One patient achieved a complete remission (CR) after the first therapy cycle, whereas the other two patients had progressive disease (PD) on day 28 and died shortly thereafter. In the responding patient, therapy was continued with irradiation and autologous hematopoietic stem cell transplantation (HSCT). One month later, this patient relapsed with Burkitt leukemia and was re-treated with blinatumomab. A second blinatumomab-induced MRD-negative CR was achieved, and blinatumomab cycles for consolidation and maintenance were added. At the last follow-up after 18 months, this patient was in continuous MRD-negative CR. AEs were CRS (highest grade 2) and neurotoxicity (highest grade 3). The authors suggested that blinatumomab can be applied safely and has activity in some r/r Burkitt lymphoma/leukemia patients [17].

Given the limited experiences with blinatumomab in r/r BL and lack of registration for this situation [12], our multi-center case series intends to expand the knowledge on the safety and efficacy of monotherapy with blinatumomab in r/r BL patients.

## 2. Materials and Methods

### 2.1. Patients

This is a multi-center retrospective and descriptive case series, analyzing data of patients with an r/r BL diagnosis who were treated with blinatumomab for relapsed or refractory disease. Patients had to be at least 18 years old and had to have relapsed or refractory BL after at least one first-line therapy. Relapse and refractoriness were evaluated according to bone marrow biopsy and PET-CT. No upper limit of prior therapy lines was set for inclusion, and no specific requirements for types of prior treatments were determined. All patients were described as one group without a control or comparison group. Follow-up was conducted until death or February 2022, whichever was first. All data were collected by the authors from December 2021 until February 2022 from chart review using EMR. Detailed characteristics of included patients at diagnosis are summarized in Table 1.

### 2.2. Treatment

All patients were treated off-label, according to the manufacturer’s instructions. Dosing schedule was identical to the phase 2 study, investigating the dosing of blinatumomab in patients with diffuse large B-cell lymphoma. A weekly stepwise dose escalation, until the target dose was reached (9, 28 and 112 mcg/day) and was preferably used instead of flat dosing (112 mcg/day from day one) [18]. One cycle of blinatumomab lasted 28 days (d) and started with 9 mcg/d in the 1st week, then the dose was increased to 28 mcg/d in the 2nd week, and in the 3rd week the target dose of 112 mcg/d was reached. This therapy scheme was the planned treatment in all patients of this cohort. Due to its short half-life, blinatumomab was administered as a continuous intravenous infusion via portable pumps [12]. The blinatumomab treatments of this cohort occurred between January 2018 and August 2021.

### 2.3. Assessment of Safety and Efficacy

Safety was evaluated according to the frequency of infection, CRS, and neurotoxicity, and the severity of CRS and neurotoxicity was graded from 1–5. Efficacy was evaluated for the endpoints overall response rate (ORR), OS, and PFS since the start of blinatumomab therapy. The response to blinatumomab was evaluated according to bone marrow biopsy, as well as radiologic assessment, preferably PET-CT, using Lugano criteria. Data on safety and efficacy were collected from medical reports.

### 2.4. Measurements and Definitions

Initial staging of patients was according to the Ann Arbor classification, and the International Prognostic Index (IPI) was determined for risk assessment [19,20]. Bulky disease was defined as lymphoma size >10 cm or mediastinal width >1/3 of chest diameter. Extranodal involvement was evaluated according to PET-CT, and infiltration of bone marrow and central nervous system (CNS) were assessed by biopsy or puncture of cerebrospinal fluid, respectively. The grading of CRS (grade 1–4) and neurotoxicity (grade 1–4) was obtained according to the ASTCT consensus grading for CRS and ICANS [21].

OS was defined as the duration from the start of blinatumomab therapy until death of any cause or last follow-up, whichever occurred first. PFS was defined as the duration from start of blinatumomab therapy until progression/relapse of disease or last follow up, whichever occurred first. The overall response rate (ORR) comprised patients achieving a complete remission (CR) or partial remission (PR) of disease following blinatumomab treatment.

### 2.5. Statistical Analysis

OS and PFS were evaluated according to Kaplan–Meier and their graphical representation was generated with GraphPad Prism^®^ (San Diego, CA, USA). Statistical calculations were made with Microsoft Excel and data of median was rounded to whole numbers. Data cut-off was on 4 March, 2022.

## 3. Results

### 3.1. Patient Characteristics at First Diagnosis

At the University Hospital Bern, Switzerland, five patients met the criteria for inclusion. The study cohort was enlarged with four patients from three other Swiss hospitals (Lausanne, Lucerne, Basel), resulting in a total number of nine patients (9/9) in the study cohort. Patients’ characteristics at first diagnosis are summarized in Table 1. Five of nine patients (5/9) were male, four were female. The median age at first diagnosis was 33 years, ranging from 25–62 years. All nine patients had sporadic BL, and MYC translocation was identified in all patients [22,23].

According to the Ann Arbor Classification, three patients (3/9) had stage II, and six patients (6/9) had stage IV disease. Two (2/9) patients had low or low-intermediate risk, and seven patients (7/9) had high-intermediate or high risk, according to the IPI score. Five patients had ECOG 0 or 1, and four patients had ECOG 2 or 3. Eight (8/9) patients had extranodal involvement, predominantly of the spleen (6/9). The bone marrow was involved in five patients (5/9), with infiltration of >25% in all, thereby formally fulfilling the definition of Burkitt leukemia [24]. No patient had central nervous system involvement. Eight patients (8/9) had B-symptoms, and five (5/9) had bulky disease. Eight (8/9) patients had an elevated lactate dehydrogenase (LDH) (upper laboratory normal limit >480 U/L), ranging from 689 to 11′276 U/L; five (5/9) had anemia (hemoglobin <100 g/L); six (6/9) had thrombocytopenia (thrombocytes <100g/); two (2/9) had leukocytosis (leukocytes <10 G/L); and in three patients (3/9) circulating peripheral Burkitt cells were identified, ranging from 4 to 72%.

### 3.2. Previous Lines of Treatment before Blinatumomab

The treatment given before blinatumomab is summarized in Table 2. Five patients (5/9) had one, two patients (2/9) had two, and two patients (2/9) had three different lines of treatment before blinatumomab. The median number of previous treatment lines was one (range 1-3); all patients had previous rituximab exposure.

The best result of first-line therapy was CR in three patients (3/9) and PR in six (6/9) patients. The disease subsequently relapsed in seven patients (7/9) and was refractory to therapy in two patients (2/9). The median duration from start of first-line therapy to relapse/refractoriness was 5 months, with a range from 3 to 11 months.

### 3.3. Blinatumomab Therapy

None of the previous treatment lines achieved a longer remission duration; thus, blinatumomab was given in rapidly progressive patients, reflecting the aggressive nature of the disease. Eight patients (8/9) started blinatumomab therapy in the setting of relapsed/refractory disease with progressive Burkitt lymphoma. In one patient, 2nd line chemotherapy led to a clearance of BL in the bone marrow with persistent disease documented by PET-CT in lymph nodes and spleen.

As planned, blinatumomab therapy was started at a dose of 9 mcg/d in all nine patients. In seven patients (7/9), the dose was increased to 28 mcg/d in week 2 of therapy, and in six (6/9) patients the target dose of 112 mcg/d was reached in the third week. The reason for not proceeding to the highest dose level in these two patients was the progression of the disease and subsequent initiation of palliative treatment in five patients, whereas dose was maintained in one patient at 28 mcg/d, based on decision of the treating physician, although blinatumomab was well tolerated. In one patient, two episodes of neurotoxicity led to a dose reduction from 112 mcg/d to 56 mcg/d, firstly, and then to a reduced dose of 28 mcg/d.

The duration of blinatumomab therapy was adapted to the individual situation (response to blinatumomab, disease progression, planned consolidation therapy), which led to administered cycles ranging from one to five cycles. Due to the rapid progression of the disease, the first cycle of therapy needed to be stopped in two patients. The median of given blinatumomab cycles was three, and except for one cycle in one patient (42 days), all cycles lasted 28 days. In patients with more than one cycle (6/9), there was a preplanned two-week therapy-free interval between cycles. During blinatumomab, two patients received additional radiotherapy.

Blinatumomab was stopped in three patients (3/9) when allogeneic HSCT was started and because of the progressive disease in the other six patients. Data on blinatumomab therapy are summarized in Table 3.

### 3.4. Adverse Effects Infection, CRS and Neurotoxicity

Infections during blinatumomab treatment occurred in four (44%) patients. Identified pathogens and manifestation are listed in Table 4. All infections were manageable with adequate antibacterial or antiviral therapy.

CRS was observed in five (5/9) patients. The severity of CRS was classified as grade 1 in one patient (1/9) and grade 2 in the remaining four patients (4/9). One patient had two episodes of CRS, both with a severity of grade 2. According to the grading, all patients had fever (body temperature ≥38°C) with/without constitutional symptoms, and those with grade 2 additionally had hypoxia (requiring low-flow oxygen delivery (≤6 L/min)), and/or hypotension (not requiring vasopressors) related to CRS [21]. No patient had severe CRS (≥ grade 3). In all five affected patients, CRS led to hospitalization, but no ICU (intensive care unit) admission was required. All patients with CRS were treated with steroids, and blinatumomab infusion was temporarily interrupted until symptoms resolved completely. Three patients (3/9) with CRS grade 2 received tocilizumab.

The symptoms of neurotoxicity caused by blinatumomab occurred in three (3/9) patients, whereas one patient had two episodes of neurotoxicity. Considering all four episodes in the three patients, three were grade 1 and one episode was grade 2. The symptoms of grade 1 neurotoxicity were aphasia, headache and tremor, and in grade 2 they were neurotoxicity dysarthria, emesis, paresthesia, headache, apraxia/ataxia and weakness. The grade 2 neurotoxicity required hospitalization but no ICU admission. Neurotoxicity was treated with a temporary interruption of blinatumomab infusion until symptoms resolved, and two out of the three patients received steroids. In all three patients, the symptoms of neurotoxicity were completely reversible. Data on adverse effects are depicted in Table 4.

### 3.5. Outcome of Blinatumomab Therapy

Three patients (3/9) achieved a CR in the bone marrow and PET-CT as best response, and two (2/9) had a PR with either PET Deauville score >3 or persisting disease in the bone marrow. CR in bone marrow was determined by morphologic bone marrow assessment (biopsy and aspirate). Unfortunately, no flow-cytometric MRD assessments were available in these three patients. In four patients (4/9), BL was progressive during blinatumomab therapy. Adding up the patients presenting with CR or PR, the ORR to blinatumomab was 5/9 patients. At the end of blinatumomab therapy, two patients (2/9) had achieved second CR, one patient (1/9) was in PR and six (6/9) had progressive disease.

**Figure 1:**
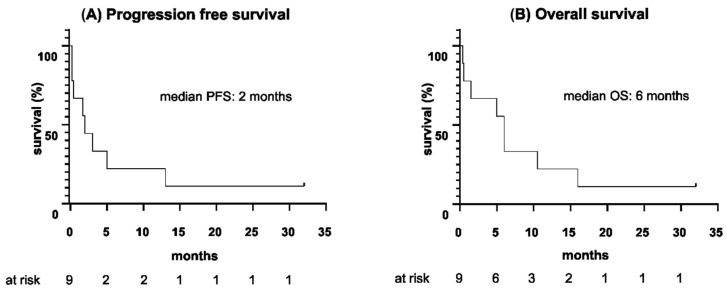
(**A**) Progression-free survival and (**B**) overall survival since the start of blinatumomab therapy (months).

Eight patients (8/9) in this cohort relapsed during or after the completion of blinatumomab therapy, with the median duration from the start of blinatumomab until relapse being 2 months (range 5 days to 13 months). The median PFS for the entire cohort was 2 months, ranging from 5 days to 32 months. All eight relapsed patients (8/9) died: seven (7/9) due to progression of disease and one (1/9) of acute liver failure, related to graft-versus-host disease (GvHD) after allogeneic HSCT. Outcomes of the individual patients are summarized in Table 5. The median OS of the cohort following initiation of blinatumomab was 6 months, with a wide range from 11 days to 32 months. PFS and OS data are provided in Figure 1 and Table 6.

**Figure 2:**
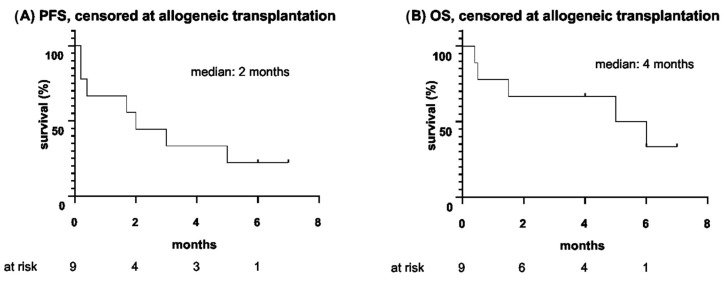
(**A**) Progression-free survival and (**B**) overall survival since the start of blinatumomab therapy, censored at allogeneic hematopoietic stem cell transplantation (months).

At the end of blinatumomab therapy, four patients (4/9) underwent allogeneic HSCT. Accordingly, Figure 2 provides survival outcomes censoring patients at the time of allogeneic HSCT. Hereby, OS was defined as the time from the start of blinatumomab until death or allogeneic HSCT, whichever occurred first, and PFS was defined as the time from the start of blinatumomab until relapse/progression of disease or allogeneic HSCT, whichever occurred first.

In eight patients (8/9), the follow-up was terminated by death, and in the 9th patient it ended in December 2021 at last follow-up, 32 months after start of blinatumomab. At this time, this patient was still in CR. The median duration of follow-up since the start of blinatumomab was 6 months (range 11 days to 32 months).

### 3.6. Treatment after Blinatumomab Therapy

Five patients (5/9) received one line of further therapy after the end of blinatumomab. One patient had radiotherapy and four patients (4/9) had allogeneic HSCT. At the time of allogeneic transplantation, two patients were in CR, one in PR and one had PD. All four patients received BEAM-Flu without TBI as conditioning regimen and immunosuppression was based on cyclosporine/steroids. Two more patients had been planned to undergo allogeneic HSCT, but relapse prevented these patients from transplant. Four relapsing patients (4/9) had no further therapy after blinatumomab except palliative symptomatic treatment. The final outcome of the treatments after blinatumomab was unfavorable. Three of the four patients with allogeneic HSCT relapsed and either died from progression of disease (two patients) or from acute liver failure related to GvHD after allogeneic HSCT (one patient). The one patient with radiotherapy after blinatumomab died from the progression of the disease. One patient with allogeneic HSCT is still alive and had been relapse-free for 32 months at the last follow-up. These data are summarized in Table 7.

## 4. Discussion

So far, there has been no effective and standardized treatment for r/r BL. Discussed options are salvage chemotherapy; autologous or allogeneic HSCT, PI3K-, CDK6- and MYC-inhibitors; and CAR T-cells [8,9,10]. The monoclonal antibody blinatumomab may provide another option for this situation. Blinatumomab has been extensively investigated in the B-ALL settings, where its efficacy for the r/r and MRD-positive situation was confirmed by many studies [25,26,27,28,29,30]. In contrast, data on its application in the r/r BL setting are scarce, with one study describing the in vitro activity of blinatumomab against BL cell lines and one report of three r/r BL patients treated with blinatumomab [16,17]. Aiming to improve insights in the potential of blinatumomab for this indication, we here retrospectively analyzed data in nine patients with a focus on data of safety and efficacy.

Overall, the tolerability of blinatumomab, administered in a weekly dose escalation (1st week 9 mcg/day, 2nd week 28 mcg/day) until the target dose of 112 mcg/day (3rd week) was reached, was favorable, and no toxicological mortalities occurred. The administration of blinatumomab via portable pumps worked without complications and could be performed mostly in an outpatient setting. Most patients of the cohort (6/9) experienced at least one of the AEs infection, CRS, and neurotoxicity, as well as 2/9 showing all three. CRS was the most frequently observed AE (5/9). A third of patients (3/9) suffered from none of these adverse effects; however, it must be considered that 2/9 received blinatumomab for only 7 days, whereas one cycle usually lasts 28 days. Interestingly, the only patient receiving blinatumomab for a longer period (12 weeks) and with none of the adverse effects assessed was the one who never received the target dose of 112 mcg/d, instead only receiving 28 mcg/d as the highest dose. In one patient, there was a need for dose reduction from 112 mcg/day to 56 mcg/day, firstly, and then 28 mcg/day due to two episodes of neurotoxicity with aphasia in the first episode and then emesis, paresthesia, headache, apraxia/ataxia and weakness in the second episode. No patient experienced CRS or neurotoxicity higher than grade 2, so there were no high-grade (≥ grade 3) AEs.

The ORR to blinatumomab in r/r Burkitt lymphoma was encouraging with 5/9 patients responding in this study. In more detail, there were 3/9 with CR and 2/9 with PR, whereby the best response was observed in patients with infiltration of the bone marrow (4/9 with CR or PR). Thus, blinatumomab showed the highest activity in patients with Burkitt leukemia in our cohort. However, it must be mentioned that in 4/9 patients, no response to blinatumomab was seen and that responses were mostly transient, with 4/5 responders relapsing within 2–13 months after the start of blinatumomab. Thus, when the whole cohort was considered, the median PFS and OS were rather short at two and six months. Still, responders showed a longer PFS and OS, as compared to the non-responders. Strikingly, 3/9 of the cohort were successfully bridged to allogeneic HSCT, which indicates that blinatumomab may be useful to prepare patients for an early allogeneic HSCT as potential curative therapy in this adverse situation. Finally, the outcome of the patients in our cohort was poor, with only one patient surviving longer term (last follow up at 32 months). This is consistent with the adverse prognosis of patients with r/r BL observed in previous studies with different salvage chemotherapy regimens and HSCT [7,8,31]. According to our results, blinatumomab seems to have activity in patients with r/r BL, but we do not think it is suitable as a curative monotherapy approach in patients with r/r BL, especially given its possible toxicity and cumbersome mode of administration (continuous intravenous infusion), and, in light of emerging CAR-T-cells, its role in r/r BL should be questioned critically.

The observation that the individual activity of blinatumomab was very variable in this study cohort might lead to the hypothesis that response to blinatumomab depends on individual patient or disease characteristics. Since the best effect of blinatumomab was seen in patients with bone marrow infiltration >25%, blinatumomab was more appropriate for patients with Burkitt leukemia in our study, as compared to non-leukemic patients.

In the context of current research, this case series can be compared with the study by Duell et al. who also investigated the safety and activity of blinatumomab in three patients with r/r BL [17]. With nine patients, the cohort of our case series was three times larger, making it the largest study of blinatumomab in patients with r/r BL to date. Concerning safety and efficacy, the two studies agree that blinatumomab could be applied safely, that there was a high interindividual variability of response and that the activity of blinatumomab in patients with r/r BL could be observed.

The use of blinatumomab should also be compared to the only officially approved therapies for r/r BL, which is so far salvage chemotherapy (e.g., Hyper-CVAD (cyclosphosphamide, vincristine, doxorubicin, dexamethasone), (R-)ICE (rituximab), ifosfamide, carboplatin, etoposide) and EPOCH (etoposide, prednisone, vincristin, cyclophosphamide, doxorubicin). The outcomes of such approaches were analyzed by Short et al. and Cremer et al., reporting an ORR of 39% (Short et al.) and 22% (Cremer et al.) and a median OS of 3.7 m (Short et al.) [7,31]. The comparison of this quantitative data with our study is not appropriate due to the small number of patients in our study, but it can be compared qualitatively. Blinatumomab has some advantages compared to chemotherapy: it penetrates the CNS, so no additional intrathecal prophylaxis is needed; its short half-life allows AEs to be simply treated with a temporary interruption of intravenous infusion; and it can be applied via portable pumps in an outpatient setting, thus guaranteeing a higher quality of life [11,12,32].

Since the number of patients in this cohort was small, no definitive conclusions should be drawn. However, considering the limited experience with blinatumomab in r/r BL in the previous literature, this study makes an important contribution to the amount of available data. Given the rarity of BL, and even more so in the refractory or relapsed situation, multi-center and international collaboration efforts would be needed to investigate blinatumomab in r/r BL in a representative number of patients.

## 5. Conclusions

The AEs infection, CRS and neurotoxicity occurred in a relevant proportion of patients (infections 4/9, CRS 5/9 and neurotoxicity 3/9) but no AE was severe (highest was grade 2), and all were treatable and reversible. Therefore, in our study, we rate the use of blinatumomab in r/r BL to be safe. The response to blinatumomab and duration until relapse and death varied widely among patients in the cohort, but with an ORR of 5/9 patients and 2/9 achieving a CR at the end of blinatumomab, the activity of blinatumomab as monotherapy in r/r Burkitt lymphoma/leukemia was clearly documented in this study. Blinatumomab proved valuable for bridging patients (3/9) to allogeneic HSCT as consolidation therapy. Unfortunately, the outcome of our cohort was unfavorable with 8/9 patients relapsing and dying within 16 months of the initiation of blinatumomab and only one patient being alive at the last follow up (32 months).

Thus, we doubt the use of blinatumomab as a curative monotherapy with long-lasting effects. Rather we see blinatumomab as a possible part of combined therapy, particularly as a bridging therapy until HSCT in curative intent. Further investigation should be made in combination with other treatments in the future by collaborative approaches, with the aim of improving the outcomes of patients with r/r Burkitt lymphoma.

## Figures and Tables

**Table 1:** Patient characteristics at first diagnosis.

Case/Characteristic	1	2	3	4	5	6	7	8	9
Age	27	33	43	52	25	50	28	30	62
Gender	m	f	m	f	m	m	m	f	f
Subtype BL	sporadic								
MYC translocation	detected								
Ann Arbor stage	IV	IV	IV	II	II	IV	IV	IV	II
IPI	High	High	High	Intermediate	Low-intermediate	High	High-intermediate	High-intermediate	High-intermediate
ECOG performance status	1	2	1	0	2	1	1	3	2
Extranodal involvement	Yes	yes	yes	no	yes	yes	yes	yes	yes
Bone marrow involvement (%)	90%	95%	45%	No	No	40%	85%	No	no
CNS involvement	No	No	No	No	No	No	No	No	no
B-symptoms	Yes	Yes	Yes	no	Yes	Yes	Yes	Yes	Yes
Bulky disease	No	No	No	Yes	Yes	No	No	Yes	yes
Peripheral blood parameters LDH (U/L) Hemoglobin (g/L) Leukocytes (G/L) Thrombocytes (G/L) Peripheral Burkitt cells (%)	982 U/L 72 g/L 17.2 (G/L) 42 (G/L) 44%	1012 U/L 68 g/L 1.2 (G/L) 24 (G/L) 0%	689 U/L 82 g/L 8.2 (G/L) 68 (G/L) 4%	712 U/L 109 g/L 5.6 (G/L) 155 (G/L) 0%	160 U/L 100 g/L 5.2 (G/L) 235 (G/L) 0%	5596 U/L 136 g/L 6.2 (G/L) 70 (G/L) 8%	11276 U/L 72 g/L 89.2 (G/L) 31 (G/L) 72%	4649 U/L 133 g/L 5.9 (G/L) 236 (G/L) 0%	1479 U/L 82 g/L 5.8 (G/L) 79 (G/L) 0%

BL: Burkitt lymphoma, IPl: international prognostic index, ECOG: Eastern Cooperative Oncology Group, CNS: central nervous system, LDH: lactate dehydrogenase.

**Table 2:** Previous lines of treatment before blinatumomab.

Case	Number of Previous Lines of Treatment	Details on Previous Lines of Treatment 1st Line 2nd Line 3rd Line	Best Response to 1st-Line Therapy	Relapse/Refractory to/after 1st-Line Treatment	Median Duration from 1st-Line Therapy until Relapse/Refractoriness (Months)
1	1	R-DA-EPOCH	CR	Relapse	8
2	1	R-CODOX-M/R-IVAC	CR	Relapse	7
3	1	R-DA-EPOCH	CR	Relapse	11
4	1	R-CODOX-M/R-IVAC	PR	Relapse	4
5	2	R-CODOX-M/R-IVAC R-DHAP	PR	Relapse	3.5
6	2	R-CODOX-M/R-IVAC CLAG-Ida-Rituximab	PR	Relapse	4.5
7	3	R-CHOP R-CODOX-M/R-IVAC CLAG-Ida-Rituximab	PR	Refractory	3
8	3	R-CHOP R-DA-EPOCH radiotherapy	PR	Relapse	6
9	1	R-CHOP	PR	Refractory	4

R-DA-EPOCH: Rituximab, dose-adjusted etoposide, doxorubicin, cyclophosphamide, vincristine, prednisone, R-CODOX-M / R-IVAC: Rituximab, cyclophosphamide, doxorubicin, vincristine / rituximab, ifosfamide, cytarabine, etoposide, R-CHOP: Rituximab, cyclophosphamide, doxorubicin, vincristine, prednisone, CLAG-Ida-Rituximab: Cladribine, cytarabine, idarubicin, rituximab, R-DHAP: Rituximab, dexamethasone, cytarabine, cisplatin, CR: complete remission, PR: partial remission.

**Table 3:** Blinatumomab therapy.

Case	Remission Status at Start of Blinatumomab	Maximum Dose Blinatumomab Given (mcg/day)	Number of Cycles of Blinatumomab	Additional Radiotherapy during Blinatumomab	End of Blinatumomab Therapy
1	PD	112	3	no	Due to progression
2	PD	112	4	No	As planned
3	PD	112	5	No	As planned
4	PD	112	2	No	Due to progression
5	PD	9	1 *	No	Due to progression
6	PR	28	3	Yes	As planned
7	PD	9	1 *	No	Due to progression
8	PD	112	3	Yes	Due to progression
9	PD	112	1	No	Due to progression

PR: partial remission, PD: progressive disease, * 2 patients received the first cycle of blinatumomab only partially.

**Table 4:** Adverse effects infection, cytokine release syndrome (CRS) and neurotoxicity.

Case	Infection, Pathogen and Manifestation	CRS	Tocilizumab Given for CRS	Neurotoxicity	Steroids Given	Hospitalization
1	Klebsiella pneumoniae, pneumonia with bacteremia	Grade 2	Yes	Grade 1	Due to CRS and neurotoxicity	Due to CRS
2	Enterobacter cloacae, bacteriemia	Grade 2	Yes	No	Due to CRS	Due to CRS
3	CMV, reactivation in colon, colitis	Grade 2	Yes	Grade 1	Due to CRS	Due to CRS
4	No	Grade 1	Yes	No	Due to CRS	Due to CRS
5	No	No	-	No	-	-
6	No	No	-	No	-	-
7	No	No	-	No	-	-
8	No	No	-	Grade 1 and Grade 2	Due to neurotoxicity	Due to neurotoxicity
9	Clostridium difficile, colitis	Grade 2	Yes	No	Due to CRS	Due to CRS

CMV: Cytomegalovirus, CRS: cytokine release syndrome.

**Table 5:** Outcome of blinatumomab therapy; presentation of data on each case.

Case	Best Response to Blinatumomab	Remission Status at End of Blinatumomab	Relapse/Progress during/after Blinatumomab	Duration until Relapse/Progression*	Death	Duration until Death *	Last Follow Up *
1	CR	PD	Yes	5 m	Due to progression	6 m	6 m
2	CR	CR	No	-	No	-	32 m
3	CR	CR	Yes	13 m	Due to progression	16 m	16 m
4	PD	PD	Yes	2 m	Due to progression	5 m	5 m
5	PD	PD	Yes	5 d	Due to progression	11 d	11 d
6	PR	PR	Yes	51 d	Due to acute liver failure GvHD-related	6 m	6 m
7	PD	PD	Yes	7 d	Due to progression	14 d	14 d
8	PR	PD	Yes	3 m	Due to progression	10 m	10 m
9	PD	PD	Yes	11 d	Due to progression	46 d	46 d

*: since initiation of blinatumomab, CR: complete remission, PR: partial remission, PD: progressive disease, d: days, m: months, GvHD: graft-versus-host disease.

**Table 6:** Outcome of blinatumomab therapy. Data on medians.

Parameter	Duration
Median duration until relapse since start of blinatumomab, m (range)	2 (5 d–13 m)
Median PFS, m (range)	2 (5 d–32 m)
Median PFS, censored at allogeneic HSCT, m (range)	2 (5 d–7 m)
Median duration until death since start of blinatumomab, m (range)	6 (11 d–16 m)
Median OS, m (range)	6 (11 d–32 m)
Median OS, censored at allogeneic HSCT, m (range)	4 (11 d–7 m)
Median follow up since start of blinatumomab, m (range)	6 (11 d–32 m)

CR: complete remission, PR: partial remission, PD: progressive disease, d: days, m: months, PFS: progression free survival, HSCT: hematopoietic stem cell transplantation, OS: overall survival, GvHD: graft-versus-host disease.

**Table 7:** Outcomes after blinatumomab therapy.

Case	Further Therapy after Blinatumomab	Status of Remission at Start of Further Therapy	Relapse/Death Following Further Therapy
1	No further therapy		
2	Allogeneic HSCT	CR	no
3	Allogeneic HSCT	CR	Relapse and death
4	Radiotherapy	PD	Relapse and death
5	No further therapy		
6	Allogeneic HSCT	PR	Relapse and death
7	No further therapy		
8	Allogeneic HSCT	PD	Relapse and death
9	No further therapy		

HSCT: hematopoietic stem cell transplantation, CR: complete remission, PD: progressive disease, PR: partial remission.

## Data Availability

The data presented in this study are available on request from the corresponding author.
